# Sirolimus-Based Immunosuppression as GvHD Prophylaxis after Bone Marrow Transplantation for Severe Aplastic Anaemia: A Case Report and Review of the Literature

**DOI:** 10.1155/2015/321602

**Published:** 2015-01-05

**Authors:** Katia Perruccio, Elena Mastrodicasa, Francesco Arcioni, Ilaria Capolsini, Carla Cerri, Grazia Gurdo, Maurizio Caniglia

**Affiliations:** Pediatric Oncology Hematology Unit, Perugia General Hospital, Località Sant'Andrea delle Fratte, 06156 Perugia, Italy

## Abstract

Congenital or acquired severe aplastic anaemia (SAA) is cured by bone marrow transplantation (BMT) from a histocompatible leukocyte antigen- (HLA-) identical sibling. The best conditioning regimen is cyclophosphamide (CTX) with or without antithymocyte globulin (ATG), followed by short-term methotrexate (MTX) and cyclosporine A (CsA) to prevent graft-versus-host disease (GvHD). In our pediatric oncology-hematology unit, a 5-year-old girl with SAA was treated with two BMT from the same HLA-identical sibling donor. Severe CsA-induced adverse events (severe hypertension and PRES) after the first BMT led necessarily to CSA withdrawal. Alternative immunosuppressive treatment for GvHD prevention as tacrolimus and mycophenolate were not tolerated by our patient because toxicity > grade II. For this reason we decided to administrate sirolimus alone as GvHD prophylaxis and to prevent disease relapse after the rescue BMT. Here we report the successful use of sirolimus alone for GvHD prophylaxis after the second transplant in a pediatric BMT setting for SAA.

## 1. Introduction

Congenital or acquired severe aplastic anemia (SAA) is a life-threatening bone marrow failure disorder whose hallmarks are pancytopenia and a hypocellular bone marrow. Mortality ensues from complications of pancytopenia [1–3], but survival has greatly improved since the advent of BMT. In fact, decision-making on therapy is guided by availability of a matched sibling donor for transplantation with ATG in the conditioning regimen and CsA as prophylaxis for GvHD [1–4]. Transplantation from alternative donors is a last resort when patients relapse or do not respond to immunosuppressive therapy.

Standard immunosuppressive therapy is a combination of horse ATG and CSA [1–3] as outcomes are not improved by adding other immunosuppressive agents like MMF [4] or sirolimus [5–9]. Few data are available on use of tacrolimus, sirolimus, and mycophenolate mofetil (MMF) as prophylaxis of graft-versus-host disease (GvHD) after BMT in SAA patients [10]. Although sirolimus was used in combination with other immunosuppressive agents for preventing GvHD after allogeneic hematopoietic stem cell transplantation (HSCT) for haematological malignancies, no data are available about its use alone or in association with other immunosuppressive agents to prevent GvHD after BMT for SAA. Here we report the case of a child with SAA who received two BMT from one HLA-identical sibling donor in whom sirolimus alone successfully prevented GvHD after the second.

## 2. Case Report

A 5-year-old girl had been affected by SAA from the age of 2, without cytogenetic abnormalities and with a bone marrow (BM) parvovirus B19 positivity, as detected by polymerase chain reaction (PCR) [11, 12]. She received a BMT from a HLA-identical sister after a conditioning regimen based on fludarabine 30 mg/m^2^/day for 4 days, CTX 300 mg/m^2^/day for 4 days, and ATG 10 mg/Kg/day for 3 days. As GvHD prophylaxis, 3 mg/Kg/day CsA from day 2 onwards and MTX (8 mg/m^2^/day on days +2, +4, and +8) were used. Engraftment occurred on day +16 after bone marrow infusion, remaining stable with 15% of host chimerism.

**Figure 1:**
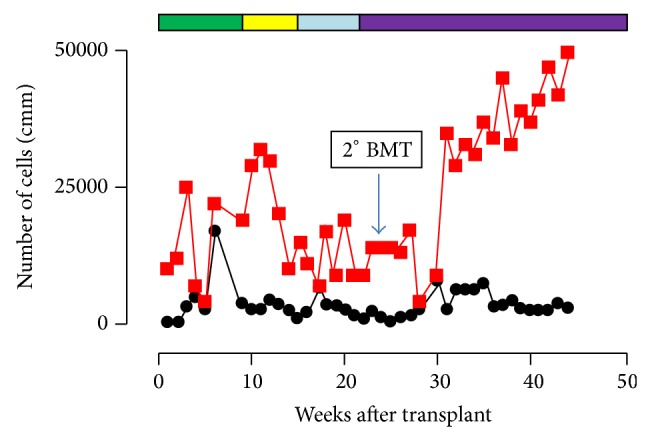
Haematological reconstitution in a 5 year old girl with SAA after first BMT (time 0) and second (22 weeks later). Black line = white blood cell counts; red line platelet counts. Coloured bars = immunosuppressive agents to prevent GvHD. Green = cyclosporine; Yellow = tacrolimus; Light blue = mycophenolate and Purple = sirolimus.

In the immediate posttransplant period, the patient developed hypertension, which was refractory to intravenous labetalol and infectious pneumonia which required broad-spectrum antibiotic therapy. On the following days, the onset of neurological symptoms led to the hypothesis of a posterior reversible encephalopathy syndrome (PRES). A CT scan of the skull showed hypodense lesions in the basal ganglia. Although CsA was suspended immediately, the child's clinical condition rapidly worsened. After repeated desaturation episodes she was transferred to the intensive care unit for assisted ventilation, dialysis, and treatment with vasoactive amines to ensure blood perfusion. MRI of the brain and spinal cord showed several anoxic-ischemic lesions in the basal ganglia, thalamus, right perirolandic region, and cervicodorsal tract of the spinal cord, indicating PRES. After flaccid paralysis for approximately one month, the patient gradually improved and slowly recovered almost all neurological functions. During this period, as GvHD prophylaxis she received mycophenolate and then tacrolimus, both of which were poorly tolerated (grade III bone marrow toxicity due to mycophenolate and grade III-IV nausea, vomiting, and weight loss due to tacrolimus). Five months after bone marrow transplant (BMT) we observed a gradual worsening of blood counts (WBC < 1,000/cmm and platelets <10,000/cmm) which reached 47% of host chimerism as shown in Figure 1. To counteract relapse the patient was given another BMT. Horse ATG 40 mg/Kg/die for 4 days was followed by infusion of cryopreserved BM stem cells from the same donor. Sirolimus replaced tacrolimus as GvHD prophylaxis because of gastrointestinal toxicity and risk of triggering PRES again.

The patient slowly and stably recovered blood counts, achieving 10–15% host chimerism. Sirolimus therapy continued as GvHD prophylaxis, modulating the dosage according to plasma levels. There was no toxicity. At present in more than 18 months after the rescue BMT, the patient is in a generally good clinical condition, with almost no neurological deficit. Blood counts are normal without transfusion. Sirolimus therapy continues to prevent GvHD and relapse of severe aplastic anaemia.

## 3. Discussion

Here we report the first case of a child with SAA who relapsed after BMT and CsA withdrawal, in whom sirolimus alone successfully prevented GvHD after a second rescue BMT. Control of GvHD was good with no toxicity. Haematological reconstitution was stable at more than one year after the second transplant.

The differential diagnosis of SAA in our young patient was between congenital and acquired SAA which share common features such as bone marrow failure and response to immunosuppressive treatments. In our patient cytogenetics were normal, thus ruling out congenital forms of AA and Fanconi's anaemia. Parvovirus positivity, the only abnormality, was eliminated by high-dose immunoglobulins before BMT. CsA had to be withdrawn because of severe PRES. Tacrolimus, the best alternative to CsA, was not tolerated by our patient because it is also a calcineurin inhibitor and caused gastrointestinal toxicity and hypertension which could have led to PRES. Therapy with MMF was not feasible during the second BMT because its myelosuppression could have interfered with engraftment and caused cytopenia. The only other option was sirolimus (a m-TOR inhibitor) which was well tolerated and successfully prevented acute and chronic GvHD, as well as relapse and graft failure.

This is the first use of sirolimus, a macrocyclic antibiotic, alone in pediatric SAA as it is commonly used in association with CsA. The synergism of sirolimus combined with CsA was established in in vitro experiments and in the clinical setting of solid organ transplantation [12]. CsA binds to calcineurin, inhibits calcium-stimulated serine/threonine phosphatase activity, and blocks T-cell activation while sirolimus blocks the multifunctional serine-threonine kinase, which is the mammalian target of rapamicine (mTOR). It also blocks CsA-resistant and calcium-independent pathways late in the T-cell cycle [12, 13] and complements CsA activity by inhibiting autoreactive cells which may escape inhibition through calcium-independent (or CsA-resistant) pathways [12].

In SAA use of sirolimus was explored by Scheinberg et al. [6] who, in a randomized study, added it to horse ATG and CsA (the standard SAA immunosuppressive treatment) in an unsuccessful attempt to elicit a better response rate. On the other hand, Young et al. reported two cases of aplastic anemia (AA) which relapsed after a CsA and ATG treatment but were successfully treated with a combination of CsA and sirolimus [11].

In the present case study since sirolimus alone has successfully prevented GvHD and SAA relapse to date, it might be a feasible alternative in SAA patients who do not tolerate CsA.
